# Small intestinal Crohn’s disease with hepatic portal venous gas: a case report

**DOI:** 10.1186/s40792-016-0193-y

**Published:** 2016-06-28

**Authors:** Masato Yamadera, Yoshiki Kajiwara, Eiji Shinto, Ryota Hokari, Hideyuki Shimazaki, Junji Yamamoto, Kazuo Hase, Hideki Ueno

**Affiliations:** Department of Surgery, National Defense Medical College, Tokorozawa, Saitama 359-8513 Japan; Department of Internal Medicine, National Defense Medical College, Tokorozawa, Saitama 359-8513 Japan; Department of Pathology and Laboratory Medicine, National Defense Medical College, Tokorozawa, Saitama 359-8513 Japan

**Keywords:** Hepatic portal venous gas, Crohn’s disease, Laparoscopic surgery

## Abstract

An 80-year-old man presented in another hospital with acute abdominal pain; computed tomography indicated hepatic portal venous gas (HPVG) and small intestinal thickening. He was then transferred to our hospital, where we diagnosed idiopathic inflammation and stenosis of the ileum. Because the patient’s abdominal symptoms were mild and his general condition was good, we chose to administer conservative therapy. His condition improved and we discharged him from our hospital. However, he was hospitalized again 9 days later because his abdominal pain had recurred and was worse. We performed a laparoscopic partial resection of the ileum 3 weeks after the patients’ initial presentation. Macroscopically, longitudinal ulcers were observed near the stenosis of the ileum; the segment of the small intestine that contained the ulcers was removed, and subsequent pathological findings indicated Crohn’s disease of the small intestine. The post-operative course was favorable, and the patient was discharged on post-operative day 9. Such serendipitous diagnosis of small intestinal Crohn’s disease in an elderly patient with hepatic portal venous gas is rare; to our knowledge, this is the first of such case in which laparoscopic surgery was performed.

## Background

Hepatic portal venous gas (HPVG) can occur alongside various gastrointestinal diseases and is often associated with a severe clinical course [1]. Recently, laparoscopic surgery for Crohn’s disease (CD) is increasing, and it has been shown to be a safe and practical approach in selected patients [2]. However, the report of laparoscopic surgery for CD with HPVG was not found so far as we searched. Additionally, the peak age of CD onset occurs from 15–20 years, and elderly onset is rare [3]. Herein, we describe the case of an 80-year-old man with small intestinal CD who developed small intestinal obstruction with HPVG and who underwent laparoscopic surgical treatment.

## Case presentation

An 80-year-old-man presented in another hospital with abdominal pain and nausea; he was transferred to our hospital for further examination and treatment, after HPVG was detected by enhanced abdominal computed tomography (CT). With regard to the patient’s medical history, he had previously undergone appendectomy, had been treated for pulmonary tuberculosis in the remote past, and had suffered tuberculous cervical lymphadenitis at the age of 76 years. His familial history was unremarkable. CT revealed marked HPVG, wall thickening in a part of the small intestine, and minor ascites in the pelvic space. At first admission, his body temperature was 38.8 °C, blood pressure was 138/84 mmHg, and pulse rate was 109 beats per minute (bpm). Physical examination revealed slight abdominal distention with mild tenderness of the lower abdomen; however, no signs of peritoneal irritation were found. As the patient was in good general health, we chose to treat him using conservative therapy. The next day, no HPVG was identified upon CT scanning, and his abdominal pain had diminished. He restarted oral feeding at that point, and was discharged on the tenth hospital day. We planned to follow him up as an outpatient; however, he was hospitalized again 9 days after discharge because his abdominal pain had recurred and was worse.

**Fig. 1:**
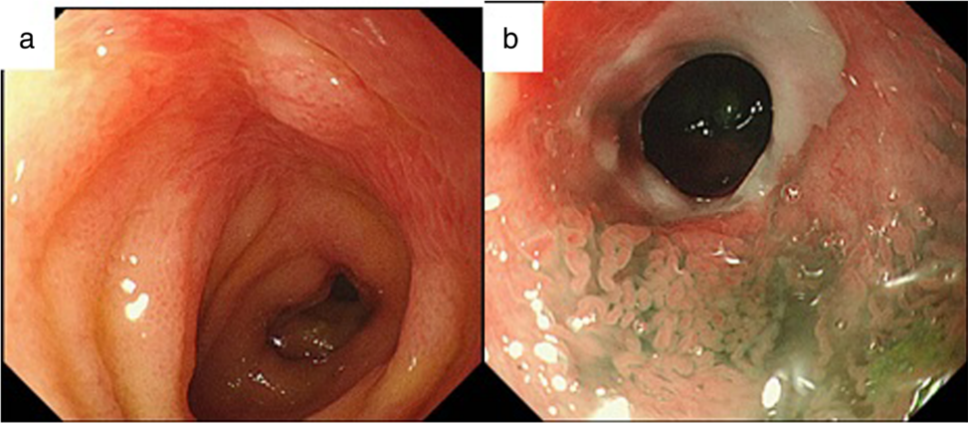
Fiber colonoscopy showing a linear ulcer (**a**) and a circular ulcer (**b**) in the ileum, 40 cm from the ileum end

**Fig. 2:**
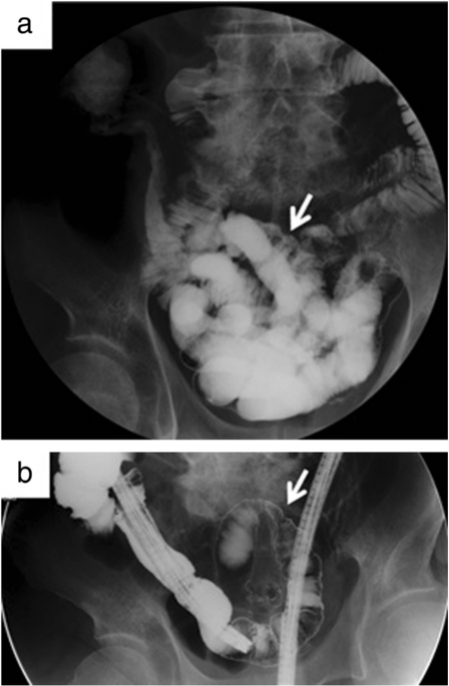
Enema examination showing the stenosis at the small intestine near the terminal ileum (**a**, **b**; *arrow*)

**Fig. 3:**
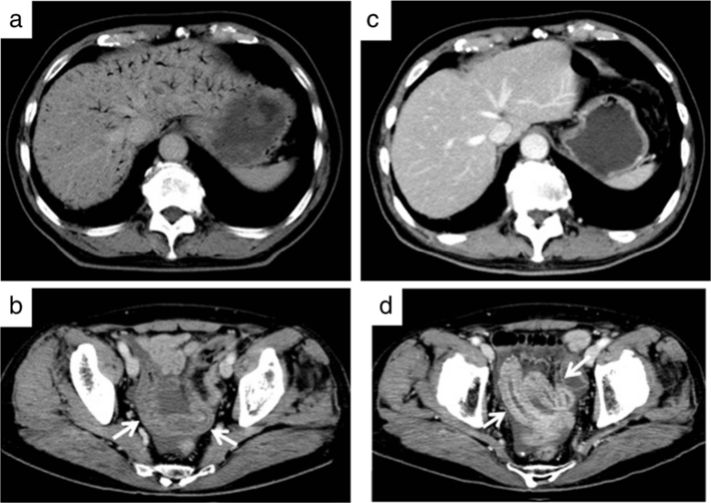
Contrast-enhanced abdominal computed tomography showing hepatic portal venous gas (HPVG) (**a**) and thickening of the ileum wall (**b**; *arrow*). Upon readmission to our hospital, the HPVG disappeared (**c**) but the ileum wall thickening did not improve (**d**; *arrow*)

At readmission, his body temperature was 37.3 °C, blood pressure was 90/56 mmHg, and pulse rate was 92 bpm. He showed slight abdominal distention and rebound tenderness of the lower abdomen, but no signs of abdominal guarding. Concerning laboratory findings, his white blood cell count was 16,800/μl, with 91.6 % neutrophils; he had a C-reactive protein level of 1.5 mg/dl. Acid-fast bacterial testing of gastric juice and sputum was negative, as was the QuantiFERON™ TB-2G test for tuberculosis. Plain radiographs of the abdomen showed the note gas in the small intestine, and colonoscopic examination revealed a linear and a circular ulcer in the ileum approximately 40 cm orally from the Bauchin valve; a stricture prevented further passage of the scope (Fig. 1a, b). Gastrografin examination of the small intestine showed constriction of the terminal ileum (Fig. 2a, b), while the jejunum was normal. Enhanced abdominal CT at readmission revealed that the HPVG (Fig. 3a, b) had not recurred (Fig. 3c); however, the thickening of the ileum wall, the edematous change, and the minor ascites in the pelvic space had not improved (Fig. 3d). No intraperitoneal air was found.

**Fig. 4:**
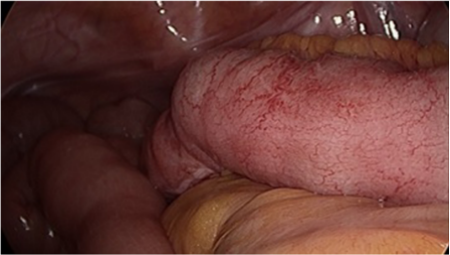
Operative findings showing inflammatory thickening of the small intestine 30 to 50 cm from the terminal ileum

**Fig. 5:**
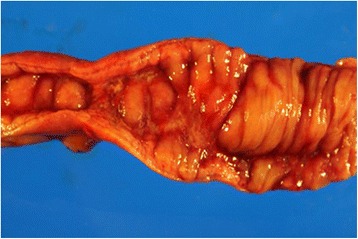
Macroscopic appearance of the resected specimen. The intestine wall was remarkably thickened. Linear ulcer and stenosis were observed

**Fig. 6:**
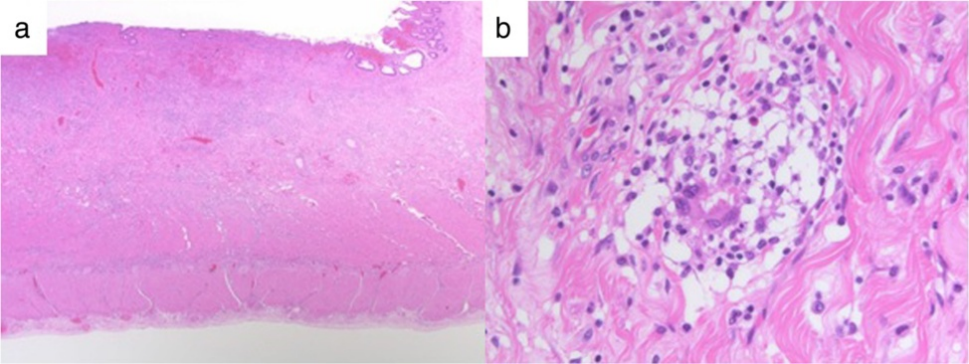
Histopathological analysis showing inflammatory cell invasion through all layers of the intestine (**a**; hematoxylin and eosin stain ×5) and epithelioid granulomas (**b**; hematoxylin and eosin stain, object lens ×40)

We presumptively diagnosed idiopathic partial ileitis with stenosis; prompted by the unresponsiveness to conservative therapy, we performed surgery. Upon laparoscopy, we found minor serous ascites and wall stiffness at the 20-cm segment of the mid-to-distal ileum. A 25-cm segment of the small intestine that included the disease was resected laparoscopically (Fig. 4). There were no adhesions throughout the peritoneal space, and the other parts of bowel were normal. The resected specimen had a thickened small intestinal wall. Linear ulcers were found approximately 5 cm on either side of the stricture, which was located at the mesentery side of small intestine. The dissection margin was free of ulceration. *Pneumatosis cystoides intestinalis* was not found (Fig. 5). Pathological examination revealed that the submucosal layer alongside the linear ulcer contained epithelioid cell granuloma. Furthermore, inflammation spanned the entire depth of the intestinal wall, with focal infiltration of neutrophils, lymphocytes, and plasma cells; this strongly indicated CD (Fig. 6). After surgery, the patient had an uneventful recovery and was discharged on post-operative day 9. He has continued to do very well following his discharge. Follow-up gastroscopy and colonoscopy have revealed no CD-like lesions in any other part of the gastrointestinal tract, from the esophagus to the anus.

### Discussion

HPVG is characterized by linear or dendric radiolucencies along the periphery of the liver on CT images. This is in contrast to biliary air, which is central and almost never extends to the periphery. Gas in the portal venous system is likely transported to small peripheral branches in the liver as a result of the centrifugal flow of the portal venous blood [4]. With regard to the mechanism of HPVG, it has been suggested that elevated intraluminal pressure caused by constriction of the intestine can allow bowel gas to enter the portal venous circulation through either microscopic mucosal perforations or disease invasions deeper than the submucosal layer [1]. HPVG has been reported in various abdominal diseases, namely, gastric ulcer, inflammatory bowel disease, bowel ischemia, acute enteritis, and simple bowel obstruction. Occasionally, the symptom is iatrogenic [5–7]. HPVG is a critical sign, with an associated mortality rate of 25 to 39 % [6–8]. Therefore, the condition has been considered an indication for urgent surgical intervention, especially if the HPVG was caused by bowel necrosis, abscess, or massive damage to the mucosa of the alimentary canal [6]. Conversely, Lim et al. [9] suggested that treatment of patients with HPVG should be based on the underlying disease, as well as the patient’s clinical condition. Moreover, Venugopal et al. [10] reported that HPVG does not necessarily indicate necrotic bowel in patients with CD; Kinoshita et al. [6] demonstrated that approximately 4 % of HPVG cases occur alongside CD and that such cases almost always have a good clinical course. Our case corroborates this claim.

HPVG is likely to occur in the elderly [6, 11]. In contrast, CD usually begins in young people—only around 5 % of CD cases have an age of onset of 65 years or older [3]. For this reason, in the present case, we suspected intestinal tuberculosis, intestinal ischemia, or post-operative adhesive small bowel obstruction rather than inflammatory bowel disease, including CD. However, repeat examinations for tuberculosis were all negative, and some macroscopic findings indicated CD in the resected specimen. Ultimately, we diagnosed CD based on histopathological findings in the resected specimen.

CD with HPVG has been reported in the literature; we carried out a search of PubMed wherein we cross-referenced the terms “Crohn's disease” and “hepatic portal venous gas.” We found 17 cases including our own (Table 1) [4, 9, 10, 12–24]. That is, new-onset small intestinal CD in an elderly patient presenting with HPVG is rare; to our knowledge, this case was the first for which laparoscopic surgery was performed. It has been suggested that laparoscopic intervention combined with treatment is useful in cases of bowel disease that lead to HPVG if (1) no peritoneal irritation signs are noted, and (2) the patient’s physical condition is stable; these two criteria describe our case well. Moreover, partial resection of the intestine that contains the affected area is a valid, reliable therapy for HPVG in cases where it may lead to more serious disease.

**Table 1:** Reported cases of Crohn’s disease (CD) with HPVG

No.	Author	Year	Age	Sex	Symptoms	Duration between onset of CD and HPVG	Operation	Outcome
1	Pappas et al. [12]	1984	36	M	Left-sided lower abdominal pain, tenesmus, liquid stools	At onset	Conservative therapy	Alive
2	Huycke et al. [13]	1985	22	M	Abdominal pain	6 years	Ileocolic resection	Alive
3	Kirsch et al. [14]	1990	26	F	Epigastric pain, chills, nausea, vomiting	At onset	Conservative therapy	Alive
4	Venugopal et al. [10]	1990	27	F	Nausea, vomiting, fever	5 years	Partial resection of the ileum	Alive
5	Delamarre et al. [15]	1991	70	M	Abdominal pain, fever	4 years	Conservative therapy	Alive
6	al-Jahdali et al. [16]	1994	40	F	Abdominal pain, nausea	20 years	Partial resection of the ileum	Not described
7	Brandon et al. [17]	2000	59	F	Nausea, vomiting, loose stool, chills vague abdominal pain	At onset	Right hemicolectomy	Not described
8	Thethy et al. [18]	2005	58	F	Malaise, rigors, bloody diarrhea, vague perianal pain	Not described	Partial colectomy	Alive
9	Salyers et al. [4]	2007	24	M	Abdominal pain, nausea	Not described	Conservative therapy	Alive
10	Alqahtani et al. [19]	2007	26	F	Right-sided upper quadrant abdominal pain	3 years	Conservative therapy	Alive
11	Hokama et al. [20]	2009	44	M	Severe abdominal pain, vomiting	28 years	Partial resection of small intestine	Alive
12	Lim et al. [9]	2011	32	M	Lower abdominal pain, nausea, vomiting, fever	12 years	Ileocecal resection	Alive
13	Ujihara et al. [21]	2013	54	F	Abdominal pain, nausea	36 years	Conservative therapy	Alive
14	Rao et al. [22]	2013	52	F	Abdominal pain, vomiting	11 years	Elective colonic resection	Alive
15	Pinto Pais et al. [23]	2014	43	F	Abdominal pain, vomiting, fever	At onset	Conservative therapy	Alive
16	Cunningham et al. [24]	2014	27	F	Fever, rigors, abdominal pain, vomiting, diarrhea	Not described	Conservative therapy	Alive
17	Our case	–	80	M	Nausea, abdominal pain	At onset	Partial resection of the ileum	Alive

## Conclusions

We have reported the first case of small intestinal CD with HPVG treated laparoscopically.

## Consent

Written informed consent was obtained from the patient for publication of this case report and any accompanying images. A copy of the written consent form is available for review by the Editor-in-Chief of this journal upon request.

## Abbreviations

bpm, beats per minute; CD, Crohn’s disease; CT, computed tomography; HPVG, hepatic portal venous gas
